# Supplementary material for: A Comprehensive Analysis of Microflora and Metabolites in the Development of Ulcerative Colitis into Colorectal Cancer Based on the Lung–Gut Correlation Theory
Source: Molecules. 2022 Sep 8;27(18):5838. doi: 10.3390/molecules27185838 (PMC9503129; doi:10.3390/molecules27185838)

Structure

1. 3-Hydroxyanthranilic Acid

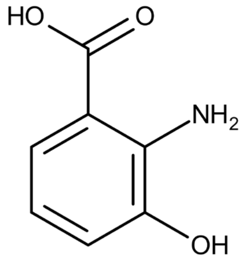

2. 3-Methylxanthine

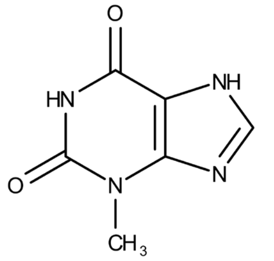

3. L-Dopa

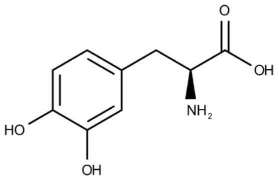

4. Oleic acid

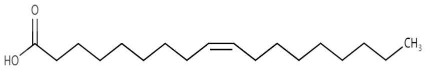

5. Tetrahydrocortisone

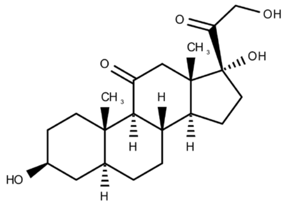

6.Isopentenyladenine-9-N-glucoside

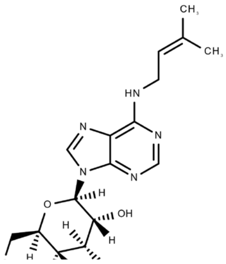

7. 4-Hydroxytamoxifen

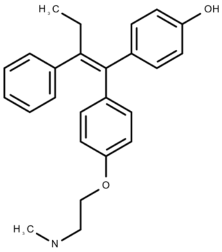

8. N-Stearoyl phenylalanine

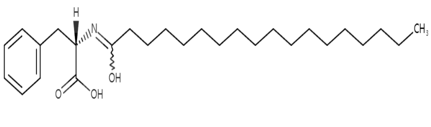

9. LysoPC(16:0/0:0)

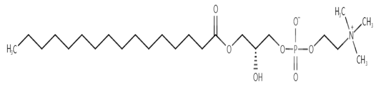

10. Taurocholic acid

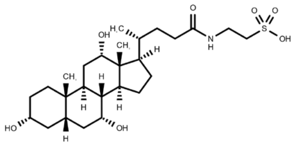

11. N-Acetylaminooctanoic acid

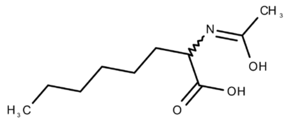

12.N-Acetylserotonin

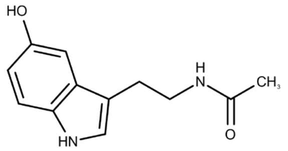

13.Tetradecanedioic acid

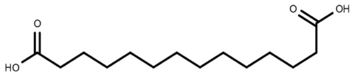

14.alpha-Linolenic acid

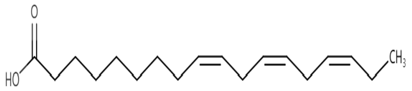

15. (9E,11E)-Octadecadienoic

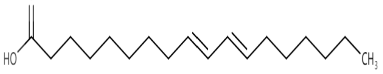

16.Guanosine

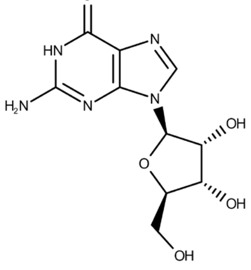

17. Oleic acid

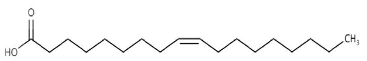

18. Stearic acid

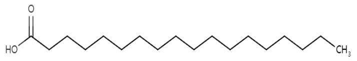

19.Hexadecanedioicacid

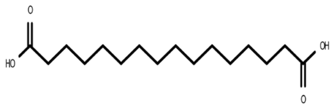

20. Eicosadienoic acid

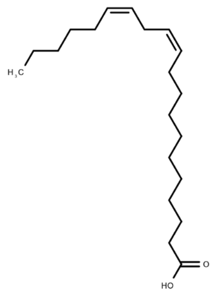

21.Tricosanoic acid

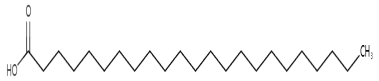

22.Tetracosanoic acid

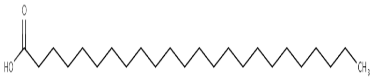

23.12b-Hydroxy-5b-cholanoic acid

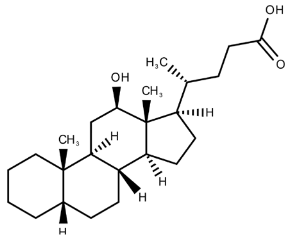

24.S-Lactoylglutathione

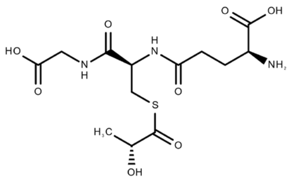

25.7-Hydroxy-3-oxocholanoic acid

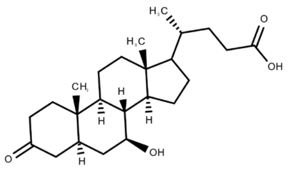

26. Succinylacetone

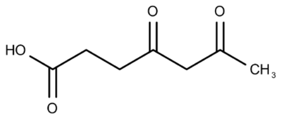

27. L-Arginine

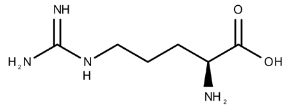

28. Hydroxykynurenine

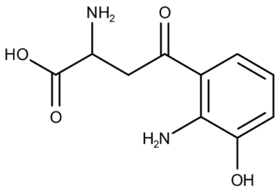

29. Deoxyinosine

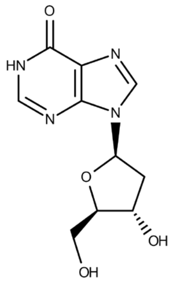

30. oleamide

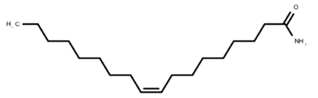

31. Sphinganine

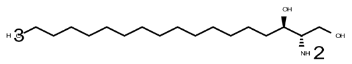

32. Phytosphingosine

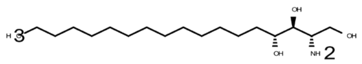

33. Tetrahydrocortisone

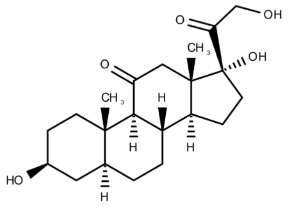

34.Tryptophan 2-C-mannoside

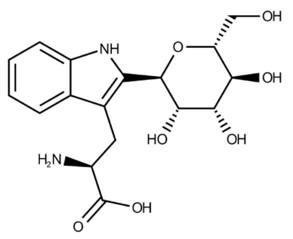

35.LysoPE (18:3(6Z,9Z,12Z)/0:0)

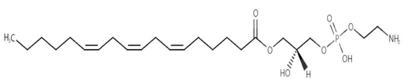

36.LysoPE (20:4(5Z,8Z,11Z,14Z)/0:0)

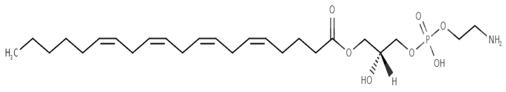

37.LysoPC (18:2(9Z,12Z)/0:0)

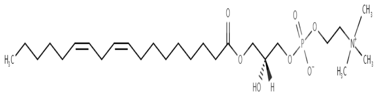

38. LysoPC (24:0/0:0)

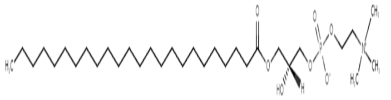

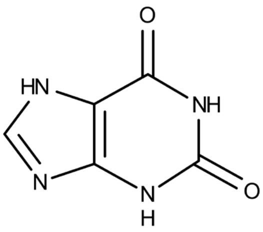

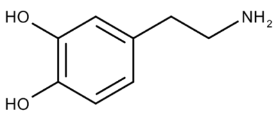

41.N-Acetylleucine

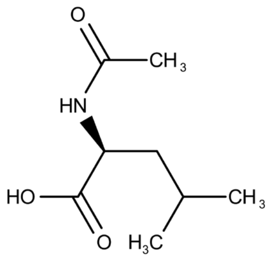

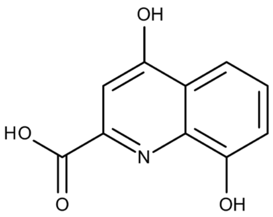

43.Tetradecanedioic acid

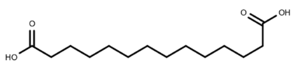

44.Adenosine

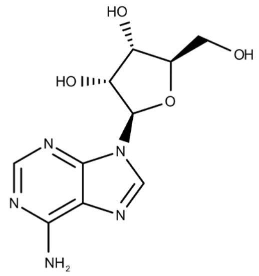

45.Heptadecanoic acid

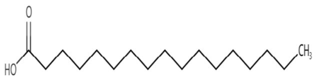

46.3-Hydroxyhexadecanoic acid

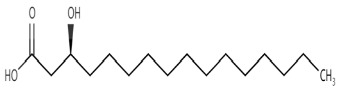

47.alpha-Linolenic acid

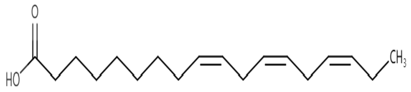

48.Tetradecanedioic acid

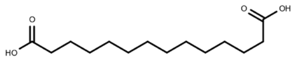

49. Oleic acid

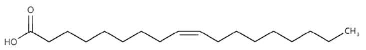

50. Xanthosine

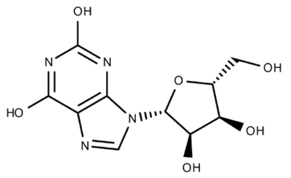

51. Hexadecanedioic acid

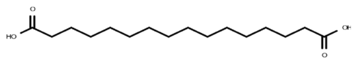

52.7-Hydroxy-3-oxochola
noic acid

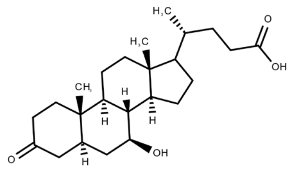

53.Deoxycholic acid

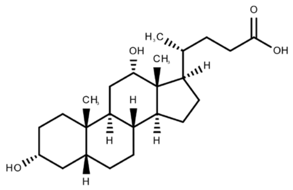

54.3a,4b,12a-Trihydroxy-5b-cholanoicac
id

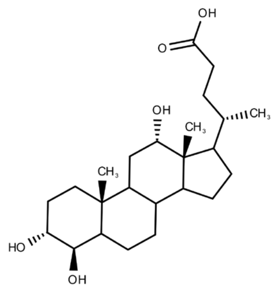

55. PE (16:0/16:0)

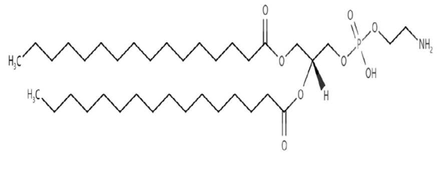

Supplement: Supplementary file 1 [file molecules-27-05838-s001.zip › molecules-1879068-Supplementary material-Structure (1).pdf]
