# Supplementary material for: A Comprehensive Analysis of Microflora and Metabolites in the Development of Ulcerative Colitis into Colorectal Cancer Based on the Lung–Gut Correlation Theory
Source: Molecules. 2022 Sep 8;27(18):5838. doi: 10.3390/molecules27185838 (PMC9503129; doi:10.3390/molecules27185838)
Supplement: Supplementary file 1 [file molecules-27-05838-s001.zip › molecules-1879068-Supplementary material-Tables and Figures.pdf]

Supplementary Tables

Table S1. Related index evaluation of Model rats during modeling process (n=8, x±SD)

Group/index	Weight(g)	DAI score[a]	CMDI score[b]	VEGF (ng/L)[c]
Control	219.6±2.8	0.04167±0.12	0.2500±0.43	81.1±3.7
Ulcerative colitis	179.2±5.6**	2.125±0.47**	3.625±0.99**	121.0±2.2**
Colorectal cancer	180.3±4.2**	2.292±0.28**	/	287.6±5.1**

*: P < 0.05, **: P < 0.01, compared with the control group. a: DAI for disease activity index. The scoring criteria for the DAI were as follows: weight score (0 point for no weight loss, 1 point for 1-5% weight loss, 2 points for 5-10% weight loss, 3 points for 10-15% weight loss, 4 points for >15% weight loss); fecal trait score (0 point for normal, 1 point for soft stool, 2 points for watery stool, 4 points for diarrhea); fecal blood status score (0 point for no blood in stool, 2 points for occult blood in stool, 4 points for apparent hemorrhage); DAI was considered the final score and was calculated using the formula, DAI = (weight score + fecal trait score + fecal blood status score)/3. b: CMDI for colonic mucosal injury index. The scoring criteria for the CMDI were as follows: 0 point for normal colonic mucosa, 1 point for mild mucosal edema, 2 points for mucosal congestion and grainy roughness, 3 points for mucosal ulcer with a diameter of 0-1 cm, 4 points for no adhesion with a mucosal ulcer of 1-2 cm, and 5 points for severe adhesion with a mucosal ulcer of 1-2 cm. c: VEGF for vascular endothelial growth factor, detected by Elisa.

Table S2. The method validation results of lung and BALF samples.

In lung													
in positive mode							in negative mode						
m/z_t_R	Precision (RSD %)		Repeatability (RSD %)		Stability (RSD %)		m/z_t_R	Precision (RSD %)		Repeatability (RSD %)		Stability (RSD %)	
	t_R	Intensity	t_R	Intensity	t_R	Intensity		t_R	Intensity	t_R	Intensity	t_R	Intensity
191.1082_9.26	0.3	10.4	1.9	3.3	0.2	2.6	171.1398_9.78	0.8	10.8	0.9	9.0	0.2	4.6
255.2326_13.96	0.2	12.4	0.2	6.9	0.3	9.2	257.2390_13.97	0.3	5.6	0.2	6.0	0.3	7.4
299.2596_11.93	0.2	6.7	0.1	9.0	0.1	11.2	279.2327_13.56	0.2	6.4	0.1	6.2	0.1	4.2
405.1895_5.20	0.1	12.7	0.1	7.4	0.1	8.8	385.2065_5.10	0.1	9.9	0.1	6.3	0.1	4.2
493.2410_5.35	0.4	13.0	0.3	9.4	0.4	6.7	449.2147_5.26	0.4	7.4	0.3	9.9	0.4	8.9
558.3333_11.77	0.2	13.2	0.1	6.5	0.2	5.2	540.3303_10.27	0.2	8.7	0.1	7.0	0.2	4.7
In BALF													
in positive mode							in negative mode						
m/z_t_R	Precision (RSD %)		Repeatability (RSD %)		Stability (RSD %)		m/z_t_R	Precision (RSD %)		Repeatability (RSD %)		Stability (RSD %)	
	t_R	Intensity	t_R	Intensity	t_R	Intensity		t_R	Intensity	t_R	Intensity	t_R	Intensity
425.2287_8.66	0.2	4.9	0.2	5.6	0.2	6.5	157.0377_3.19	1.0	12.8	4.0	14.9	0.7	12.8
432.7596_6.76	0.9	11.6	0.2	10.6	0.5	6.7	303.2320_14.74	0.9	6.5	0.9	14.7	1.2	10.0
454.7728_6.79	0.8	7.1	0.8	13.9	0.9	13.3	307.2614_15.85	0.2	12.1	0.7	13.4	0.6	13.4
476.7857_6.80	0.6	5.0	0.9	6.5	0.4	6.8	322.8546_2.98	1.1	8.0	0.3	8.8	0.7	7.6
498.7990_6.84	0.4	9.3	0.6	13.9	0.7	14.4	327.2296_14.40	0.6	9.4	1.6	14.6	0.7	13.8
518.3207_11.51	0.4	8.9	0.1	11.4	0.1	9.9	447.2741_9.85	0.6	14.7	0.8	13.6	0.4	13.1

Table S3. The statistics of Alpha diversity index values of each sample. (n=4)

Sample name	OTU	shannon	simpson	chao1	ACE	coverage
C1	393	3.397	0.766	436.784	448.887	0.999
C2	660	6.161	0.923	714.638	718.213	0.998
C3	535	3.516	0.807	604.734	619.894	0.998
C4	702	4.149	0.834	770.703	782.326	0.998
U1	590	5.527	0.944	705.957	693.551	0.998
U2	748	4.085	0.835	823.000	840.337	0.997
U3	590	4.800	0.918	660.611	679.084	0.998
U4	1077	5.971	0.927	1118.362	1119.521	0.998
R1	565	2.044	0.443	639.043	649.868	0.998
R2	775	2.511	0.460	864.013	884.086	0.997
R3	612	2.772	0.649	670.508	687.417	0.998
R4	669	2.157	0.517	761.329	780.160	0.997

OTU: Operational Taxonomic Unit; Chao1 and Ace indices measure the number of species; The Shannon and Simpson indices were used to measure species diversity (the larger the Shannon index value and the smaller the Simpson index value, the higher the species diversity of this sample was); Coverage represents the probability that the bacterial community in the sample was detected.

Supplementary Figures

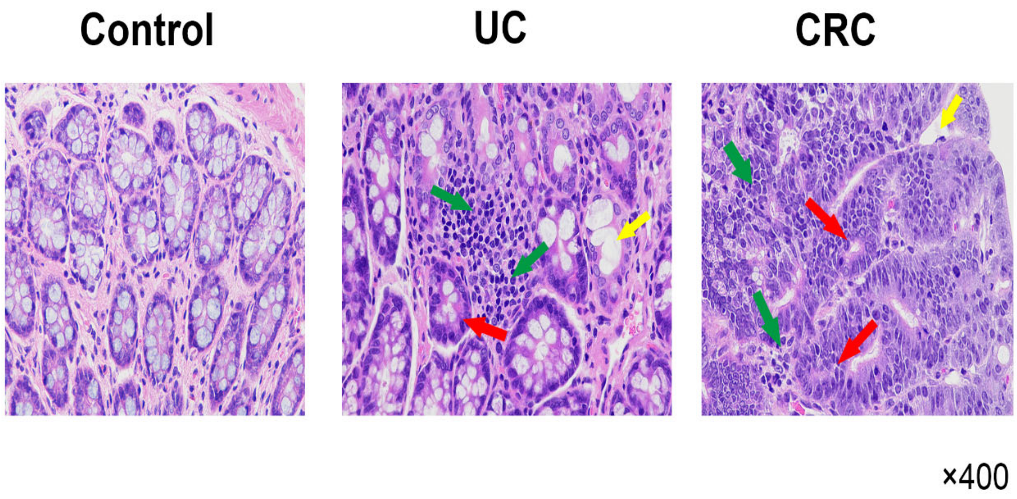

Figure S1. Histological changes in colon sections stained with hematoxylin and eosin of control, UC and CRC groups (400× magnification). The red arrows represent the loss of mucus in the goblet cells. The green arrows represent inflammatory cell infiltration; The yellow arrows represent intestinal epithelial vacuoles.

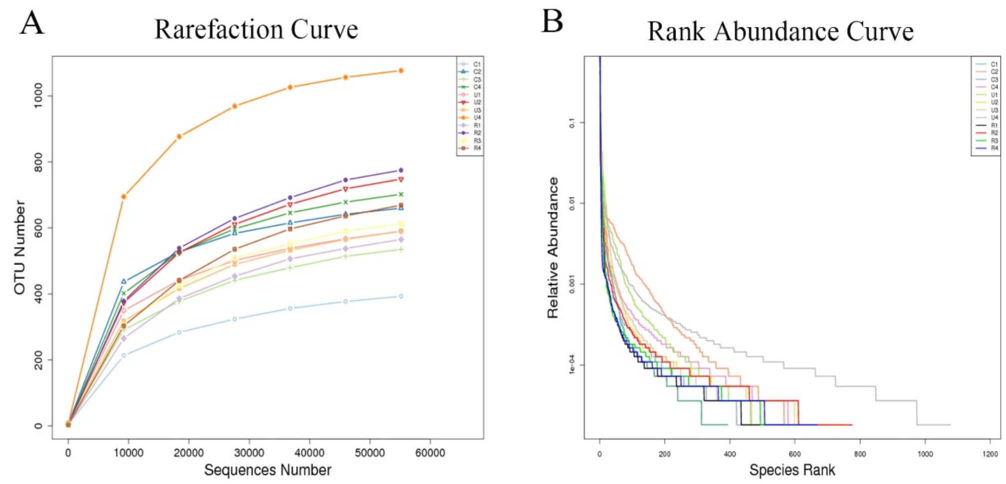

Figure S2. (A) Rarefaction Curve. (B) Rank Abundance Curve.
